# Chronic Obstructive Pulmonary Disease among Patients Admitted to the Department of Medicine in a Tertiary Care Centre

**DOI:** 10.31729/jnma.8257

**Published:** 2023-09-30

**Authors:** Milan Khadka, Lochan Karki, Shrinkhala Maharjan, Ganesh Kumar Giri, Pasang Sherpa, Poonam K C, Siddhant Adhikari, Milan Purna Oli, Rama Tamrakar, Santosh Joti

**Affiliations:** 1 Department of Medicine, National Academy of Medical Sciences, Mahaboudha, Kathmandu, Nepal; 2 Annapurna Neurological Institute & Allied Sciences, Maitighar Mandala, Kathmandu, Nepal; 3 National Institute of Neurological and Allied Science, Bansbari, Kathmandu, Nepal; 4 B&B Hospital Pvt. Ltd., Gwarko, Lalitpur, Nepal; 5 Ganeshman Singh Memorial Hospital and Research Center, Ring Road, Lalitpur, Nepal; 6 Mulpani Nagar Hospital, Kageshwori Manohara, Kathmandu, Nepal; 7 Godawari Midcity Hospital, Satdobato, Lalitpur, Nepal; 8 Lubhoo Primary Health Care Centre, Mahalaxmi, Lalitpur, Nepal; 9 Silverline Hospital, Balaju, Kathmandu, Nepal

**Keywords:** *COPD*, *inpatients*, *prevalence*, *tertiary hospital*

## Abstract

**Introduction:**

Chronic obstructive pulmonary disease is a prevalent respiratory condition with permanent and progressively decreasing airflow limitation. Chronic obstructive pulmonary disease causes more than 3 million deaths per year globally, making it the third leading cause of death globally. The aim of this study was to find out the prevalence of chronic obstructive pulmonary disease among patients admitted to the Department of Medicine in a tertiary care centre.

**Methods:**

A descriptive cross-sectional study was done in the Department of Medicine of a tertiary care centre. Data from 1 January 2022 to 30 December 2022 were collected between 15 June 2023 to 30 June 2023 from the hospital records and reviewed. Ethical approval was taken from the Institutional Review Committee. All the patients admitted to the Department of Medicine during the study period were included in the study. The patients with incomplete medical records were excluded from the study. Convenience sampling method was used. The point estimate was calculated at a 95% Confidence Interval.

**Results:**

Among 280 patients, chronic obstructive pulmonary disease was found in 68 (24.29%) (19.27-29.31, 95% Confidence Interval) with a mean age of 70.62±10.39 years and a mean pack year of 16.72±7.67.

**Conclusions:**

The prevalence of chronic obstructive pulmonary disease among patients admitted to the Department of Medicine was similar to the other studies done in similar settings.

## INTRODUCTION

Chronic obstructive pulmonary disease (COPD) is one of the major preventable chronic respiratory diseases.^1^ COPD patients present with a variety of respiratory symptoms such as dyspnea, coughing, and/or sputum production.^2^ Cigarette smoking is the major risk factor for COPD globally, occupational exposure and indoor air pollution are additional risk factors for COPD.^3^

COPD is one of the leading causes of chronic morbidity and mortality worldwide. Many people experience years of suffering before death from COPD or its complications.^4^ COPD causes more than 3 million deaths per year globally, making it the third-leading cause of death globally.^4,5^

The aim of this study was to find out the prevalence of chronic obstructive pulmonary disease among patients admitted to the Department of Medicine in a tertiary care centre.

## METHODS

This descriptive cross-sectional study was conducted among admitted patients in the Department of Medicine at the National Academy of Medical Sciences (NAMS), Mahaboudha, Kathmandu, Nepal. Ethical approval was obtained from the Institutional Review Committee of the same institution (Reference number: 778/2079/80). Data from 1 January 2022 to 30 December 2022 were collected between 15 June 2023 to 30 June 2023 from the hospital records. All the patients admitted in the Department of Medicine within the study period with complete hospital records were included in the study. The patients with incomplete medical records were excluded from the study. Convenience sampling method was used. The sample size was calculated by using the following formula:


$$\begin{array}{ll} \text{n} & ={\text{Z}}^{2}\times \frac{\text{p}\times \text{q}}{{\text{e}}^{\text{2}}}\\ & =1.96^{2}\times \frac{0.50\times 0.50}{0.08^{2}}\\ & =150 \end{array}$$

Where,

n = minimum required sample size
Z = 1.96 at 95% Confidence Interval (CI)
p = prevalence taken as 50% for maximum sample size calculation
q = 1-p
e = margin of error, 8%

The minimum required sample size was 150. However, the final sample size taken was 280.

Patients who displayed FEV1/FVC 0.70 or below LLN cut-off values in the post-bronchodilator test with the presence of symptoms were classified as having COPD according to the Global Initiative for Chronic Lung Disease (GOLD) criteria.^7^

Data was extracted using the extraction sheet using Microsoft Excel 2016 and analysed using IBM SPSS Statistics version 26.0. The point estimate was calculated at a 95% CI.

## RESULTS

Among 280 patients, COPD was found in 68 (24.29%) (19.27-29.31, 95% CI) with a mean age of 70.62±10.39 years. The mean hospital stay was found to be 6.82±5.10 days. A total of 43 (63.24%) patients were female (Table 1).

**Table 1:** Demographic characteristics of the patients with COPD (n= 68).

Characteristics	n (%)
**Gender**	
Male	25 (36.76)
Female	43 (63.24)
**Age group (years)**	
30-44	2 (2.94)
45-59	6 (8.82)
60-74	37 (54.41)
75-89	21 (30.88)
≥90	2 (2.94)
**Ethnicity**	
Chettri	20 (29.41)
Newar	12 (17.65)
Brahmin	9 (13.24)
Magar	6 (8.82)
Others	21 (30.88)
**Residency**	
Rural	47 (69.12)
Urban	21 (30.88)

Among 68 COPD patients, 61 (89.71%) patients were= 150smokers with a mean pack year of 16.72±7.67. A total of 35 (51.47%) patients wereexposed to indoor airpollution (Table 2).

**Table 2:** History of smoking and indoor air pollution among patients with COPD (n= 68).

Exposure	n (%)
Smoking	61 (89.71)
Indoor air pollution	35 (51.47)

**Figure 1:**
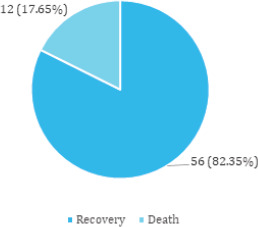
Outcome among the COPD patients (n= 68).

Among COPD patients, 10 (14.71%) required ICU admission whereas 58 (85.29%) were treated in the medicine ward. A total of 56 (82.35%) patients recovered and were discharged whereas 12 (17.65%) died in the hospital (Figure 1).

Among COPD patients, hypertension was seen as the most common comorbidity with 22 (32.35%) patients having the condition followed by COVID-19 which was 15 (22.05%). Among 68 COPD patients, cor pulmonale was seen in 18 (26.47%) patients as a COPD complication (Table 3).

**Table 3:** Comorbidities and complications among COPD patients (n= 68).

Comorbidities	n (%)
Hypertension	22 (32.35)
COVID-19	15 (22.06)
Type 2 diabetes mellitus	7 (10.29)
**Complications**	
Corpulmonale	18 (26.47)
Respiratory failure	5 (7.35)
Polycythemia	5 (7.35)

## DISCUSSION

The prevalence of COPD admitted in Department of Medicine was found to be 68 (24.29%) which was similar to the study done in another tertiary care centre of Nepal which was found to be 23.17%.^6^ Another study done in the Nepal Medical College of Nepal showed that the prevalence of COPD was found to be 17.3%.^8^ However, a study done in Saudi Arabia showed that the prevalence of COPD among hospitalized patients was found to be 2.4% which is very much lower in comparison to our study.^9^ Nepal is a low-income country and has higher predisposing factors, so the prevalence of COPD seems higher.^4,5^

The majority of patients were in the age group of 6074 years with a mean age of 70.62±10 years which is slightly different than the previous study where the 71-80 age group was major and the mean age group was 73.5±2.76 years.^10^ Similarly, females with COPD were higher in number, 43 (63.24%) than males 25 (36.76%) which was similar with other study but less (52.2%) which is a contrast to other studies in global prevalence where males are higher in number.^11,12^

Hypertension was found to be the most common 22 (32.35%) comorbidity in patients with COPD which is similar to other studies done in a similar setting. However, COVID-19 disease is the second most common comorbidities in patients with COPD 15 (22.05%) in our study surpassing diabetes mellitus which was the second most in studies done before the COVID-19 pandemic.^5,13^ Cor pulmonale was found to be the most common complication of COPD 18 (26.47%) which is less than a similar study done in a similar setting which shows 68% have cor pulmonale.^3^

There were a few limitations to the study. Our study is single-centered and the sample size is also small. Therefore, the results cannot be generalized in a larger population. Also, this is a study done in a developing country so the prevalence might vary from the hospitals of developed and underdeveloped countries.

## CONCLUSIONS

The prevalence of COPD among patients admitted to the Department of Medicine was similar to other studies done in a similar settings.
